# Evaluation of Cell Lines for the Isolation of Foot-and-Mouth Disease Virus and Other Viruses Causing Vesicular Disease

**DOI:** 10.3389/fvets.2020.00426

**Published:** 2020-07-28

**Authors:** Ashley R. Gray, Britta A. Wood, Elisabeth Henry, Mehreen Azhar, Donald P. King, Valérie Mioulet

**Affiliations:** Vesicular Disease Reference Laboratory, The Pirbright Institute, Surrey, United Kingdom

**Keywords:** foot-and-mouth disease virus, virus isolation, cell line, diagnosis, vesicular viruses

## Abstract

The most sensitive cell culture system for the isolation of foot-and-mouth disease virus (FMDV) is primary bovine thyroid (BTY) cells. However, BTY cells are seldom used because of the challenges associated with sourcing thyroids from FMDV-negative calves (particularly in FMD endemic countries), and the costs and time required to regularly prepare batches of cells. Two continuous cell lines, a fetal goat tongue cell line (ZZ-R 127) and a fetal porcine kidney cell line (LFBK-α_V_β_6_), have been shown to be highly sensitive to FMDV. Here, we assessed the sensitivity of ZZ-R 127 and LFBK-α_V_β_6_ cells relative to primary BTY cells by titrating a range of FMDV original samples and isolates. Both the ZZ-R 127 and LFBK-α_V_β_6_ cells were susceptible to FMDV for >100 passages, and there were no significant differences in sensitivity relative to primary BTY cells. Notably, the LFBK-α_V_β_6_ cell line was highly sensitive to the O/CATHAY porcine-adapted FMDV strain. These results support the use of ZZ-R 127 and LFBK-α_V_β_6_ as sensitive alternatives to BTY cells for the isolation of FMDV, and highlight the use of LFBK-α_V_β_6_ cells as an additional tool for the isolation of porcinophilic viruses.

## Introduction

Foot-and-mouth disease (FMD) is a highly contagious disease of cloven-hoofed animals, which results in widespread economic burden (1). The major cause of global spread is the transboundary movement of animals, and as such, animal trade is restricted in countries where the disease is present (2). Foot-and-mouth disease virus (FMDV; family *Picornaviridae*, genus *Aphthovirus*) is the causative agent, and there are seven different serotypes [O, A, C, Asia 1, Southern African Territories (SAT) 1, SAT 2, and SAT 3; (3)], with many different topotypes within each serotype (4).

Control of FMD is underpinned by rapid and accurate diagnosis. Virus isolation using susceptible cell cultures is beneficial for the amplification of virus for downstream diagnostic tests, including FMD serotyping by antigen enzyme linked immunosorbent assay (ELISA) (5) and sequencing of the VP1 region of the genome (6). Cell cultures are also required to produce FMDV vaccines, which are currently based on inactivated whole virus preparations (7). Control of FMD through vaccination is complicated by limited cross serotype/topotype immunity and therefore, vaccine matching field isolates using susceptible cell lines is an essential tool for appropriate vaccine selection (7).

Primary bovine thyroid (BTY) cell cultures are the most sensitive system for the isolation of FMDV (8), but their use is not widespread because of the difficulties obtaining tissue, the time and expense required to prepare the cells, and the fact that the cells have a relatively short life span. Immortalized cell lines, such as baby hamster kidney fibroblasts (BHK-21) and pig kidney (IB-RS-2) cells, provide a stable source of susceptible cultures, but are generally less sensitive to FMDV (8). Nonetheless, porcine cells (e.g., IB-RS-2) are commonly required for the isolation of FMDV strains that have naturally adapted to infect pigs (9), such as the serotype O/CATHAY topotype, which do not replicate in BTY cells. For diagnostic laboratories, it is also important that cell culture systems are able to support the propagation of viruses that cause clinical disease that are indistinguishable from FMDV, such as swine vesicular disease virus (SVDV), vesicular exanthema of swine (VESV), vesicular stomatitis virus (VSV), and Seneca Valley virus (SVV).

Fetal porcine kidney (LFBK-α_V_β_6_) cells, which have been engineered to express bovine α_V_β_6_ integrin, a principal cellular receptor of FMDV, and fetal goat tongue cells (ZZ-R 127) are two continuous cell lines that are highly sensitive to FMDV (10–12). A number of studies have utilized the LFBK-α_V_β_6_ and ZZ-R 127 cell lines for the isolation of FMDV from different clinical samples (13–20). In previous studies, the ZZ-R 127 cell line provided similar sensitivity to FMDV as primary BTY cells (10) and LFBK-α_V_β_6_ cells (21), however to our knowledge the LFBK-α_V_β_6_ cell line has not been compared to BTY cells. The World Reference Laboratory for FMD (WRLFMD; The Pirbright Institute, UK) currently utilizes BTY and IB-RS-2 cells for the diagnosis of FMDV. In this study, the diagnostic capabilities of ZZ-R 127 and LFBK-α_V_β_6_ cells lines were evaluated using epithelium suspensions from a range of FMDV serotypes/subtypes, as well as the effects of different sample matrices commonly used for the isolation of FMDV. Through comparative titrations, we assessed the longevity of sensitivity of ZZ-R 127 and LFBK-α_V_β_6_ cells lines to FMDV isolates alongside BTY and IB-RS-2 cells. Finally, the ability of ZZ-R 127 and LFBK-α_V_β_6_ cells lines to propagate representative isolates of VESV, VSV, and SVV was also determined.

## Materials and Methods

All experiments were conducted at The Pirbright Institute in high-containment laboratories that meet the *Minimum Biorisk Management Standards for Laboratories Working with Foot-and-Mouth Disease Virus* of the European Commission for the Control of Foot-and-Mouth Disease (22).

### Cells

BTY cells were prepared weekly incorporating variations from the method previously described in Snowdon (23). Briefly, bovine calf thyroids were obtained from an abattoir, dissociated using dispase II (Gibco), and cultured using Eagle's Glasgow minimal essential medium (GMEM; Sigma) supplemented with 12 mL/L field antibiotics (0.002 mg/mL amphotericin B, 10^−4^ MU/mL penicillin, 49 μg/mL neomycin, 98 U/mL polymyxin B, sterile water), 10 mL/L L-glutamine (Sigma), and 10% adult bovine serum (ABS; Sigma). The BTY cells were counted using a Fuchs-Rosenthal counting chamber and the concentration normalized to a seeding density of 6 × 10^5^ cells/mL. The BTY cells were cultured in Nunc™ flat-sided cell culture tubes (5.5 cm^2^; Thermo Fisher Scientific) using 2 mL of cell suspension and incubated stationary at 37°C. After 96 h, the media was discarded from each tube and replaced with GMEM (Sigma) supplemented field antibiotics and L-glutamine as above and between 2 and 10% ABS (Sigma). The percentage of ABS used was dependent on the average level of confluency observed in 10 tubes after 96 h (e.g., <40% confluence – 10% ABS, 40–60% confluence – 7% ABS, 60–90% confluence – 5% ABS, >90% confluence – 2% ABS). After the media change, the cell culture tubes were incubated with rotation at 37°C until use.

IB-RS-2 cells were maintained in T-175 cell culture flasks using GMEM (Gibco) supplemented with 10% adult bovine serum (Sigma). The seed stocks were passaged to reach 90–100% confluency in 72 to 96 h. The IB-RS-2 cells were prepared in Nunc™ cell culture tubes using 2 mL of cell suspension at a concentration between 0.5 and 6 × 10^5^ cells/mL to reach 90–100% confluency between 24 and 96 h. Seed flasks and cell culture tubes were incubated stationary at 37°C until use.

ZZ-R 127 cells, supplied by the Friedrich-Loeffler-Institute (Greifswald-Insel Riems, Germany), were maintained in T-175 cell culture flasks using Dulbecco's modified Eagle medium: F12 (DMEM; Lonza) supplemented with 10% fetal bovine serum (Gibco). The seed stocks were passaged to reach 90–100% confluency in 96 h. The ZZ-R 127 cells were cultured in Nunc™ cell culture tubes using 2 mL of cell suspension at a concentration of 0.65 × 10^5^ cells/mL to reach 90–100% confluency in 96 h. Seed flasks and cell culture tubes were incubated stationary at 37°C until use.

LFBK-α_V_β_6_ cells (11, 12), supplied by the Animal and Plant Health Inspection Service, Diagnostic Service Section at the Plum Island Animal Disease Center (Long Island, NY, USA), were maintained in T-175 cell culture flasks using DMEM (Gibco) supplemented with 10% fetal bovine serum (Gibco). The seed stocks were passaged to reach 90–100% confluency in 72 h. The LFBK-α_V_β_6_ cells were cultured in Nunc™ cell culture tubes using 2 mL of cell suspension at a concentration of 2 × 10^5^ cells/mL to reach 90–100% confluency in 72 h. Seed flasks and cell culture tubes were incubated stationary at 37°C until use.

Preparation of primary cell cultures and passaging of continuous cell lines were performed inside a class 2 microbiological safety cabinet. Biocontainment procedures were required for the maintenance of IB-RS-2 cells and LFBK-α_V_β_6_ cells, which are persistently infected with classical swine fever (CSF) virus (24) and a non-cytopathic bovine viral diarrhea virus (BVDV; Rodriguez LL, personal communication, 2019), respectively. All virus isolations and titrations were performed using monolayers of 90–100% confluency cultured in Nunc™ cell culture tubes. All cell culture tubes received minimal essential media (MEM; Gibco) supplemented with 6 mL/L field antibiotics and 2% fetal bovine serum (Gibco) to sustain cell cultures after the addition of virus and negative matrices.

### Virus Stocks

In line with the OIE manual (25), FMDV and SVDV original suspensions were prepared by homogenizing vesicular epithelium as a 10% solution in M25 buffer (35 mM disodium hydrogen phosphate, 5.7 mM potassium dihydrogen phosphate, sterile water). The tissue was homogenized with sterile sand (Sigma) using a sterilized pestle and mortar. The suspension was clarified by centrifugation at 3,000 g for 10 min at 4°C.

Epithelial suspensions tested in the diagnostic sensitivity experiments were either used immediately after preparation or aliquoted and stored at −80°C. The suspensions of FMDV/A/IRN/24/2012, FMDV/O/KUW/4/2016 and SVDV/UKG/77/80 prepared for the longevity of sensitivity experiments were mixed 1:1 with glycerol (VWR chemicals) for long term storage at −20°C. SVV, VESV, and VSV New Jersey isolates of known high viral titers were selected from the WRLFMD virus collection.

### Virus Titrations

Virus titrations were performed in parallel to compare the relative sensitivity of the cell lines to FMDV and SVDV. Virus stocks were serially diluted 10-fold in M25 buffer. Cells (*n* = 4 or 5 tubes per cell line) were washed with 2 mL sterile phosphate buffer saline (PBS; Severn Biotech) before adding 2 mL of MEM (Gibco). The cell tubes were then inoculated with 0.2 mL of the appropriate virus dilution and incubated with rotation at 37°C for 72 h, after which the cells were visually examined under a microscope for cytopathic effect (CPE). For each cell line, viral titers were calculated using the Spearman-Karber method and expressed as Log_10_ TCID_50_/mL, where a higher viral titer in a cell line correlated to a lower limit of detection and greater analytical sensitivity.

A/IRN/24/2012 and O/KUW/4/2016 glycerinated epithelium suspensions were initially titrated with BTY cells to establish baseline titers (6.6 and 7.8 Log_10_ TCID_50_/mL, respectively). The continued sensitivity of ZZ-R 127 and LFBK-α_V_β_6_ to FMDV was assessed by titrating A/IRN/24/2012 or O/KUW/4/2016 during continued passaging of the cell lines; all titrations were performed in parallel with BTY and IB-RS-2 cells. A/IRN/24/2012 was used for 9 months (16th May 2017 to 6th February 2018) until viral titers began to decrease across all cell lines, possibly due to sample degradation, and was replaced with O/KUW/4/2016 that was used for 10 months (12th February 2018 to 18th December 2018).

SVDV/UKG/77/80 glycerinated epithelium suspension was initially titrated with IB-RS-2 cells to establish a baseline titer (3.8 Log_10_ TCID_50_/mL). The sensitivity of the LFBK-α_V_β_6_ cell line to SVDV was assessed over time by titrating UKG/77/80 during continued passaging of the cell line; all titrations were performed in parallel with IB-RS-2 cells. The ZZ-R 127 cell line was not included in these experiments because SVDV does not propagate in this cell line (10).

### FMDV Diagnostic Sensitivity

Forty epithelium suspensions (Table 1), representing five serotypes and thirteen topotypes of FMDV (O *n* = 20, A *n* = 8, SAT 1 *n* = 4, SAT 2 *n* = 3, and Asia 1 *n* = 5), were either retrieved from −80°C storage or prepared from epithelial tissue. Titrations were performed with BTY, ZZ-R 127, and LFBK-α_V_β_6_ cells for all samples, except for the O/CATHAY topotype. O/CATHAY is a porcine adapted strain and does not replicate in BTY cells. The O/CATHAY samples were titrated using IB-RS-2, ZZ-R 127, and LFBK-α_V_β_6_ cells.

**Table 1:** Number of epithelium suspensions tested by serotype and lineage.

**Serotype**	**Topotype**	**Lineage**	**Sub-lineage**	**No of isolates**
O	CATHAY	–	–	8
	SOUTH EAST ASIA	Mya-98	–	1
	MIDDLE EAST SOUTH ASIA	Ind-2001	d	2
			e	1
		PanAsia	–	1
		PanAsia2	ANT-10	1
			BAL-09	2
			QOM-15	1
	WEST AFRICA	–	–	1
	EAST AFRICA 2	–	–	1
	EAST AFRICA 3	–	–	1
A	ASIA	Iran-05	FAR-11	2
			SIS-13	2
			SIS-10	1
		G-VII	–	2
	AFRICA	G-IV	–	1
SAT 1	III	–	–	1
	III (WZ)	–	–	2
	X	–	–	1
SAT 2	VII	Alx-12	–	2
		Lib-12	–	1
ASIA 1	ASIA	Sindh-08	–	3
		–	–	2
Total				40

Twenty-six diagnostic porcine epithelium suspensions originating from Hong Kong were inoculated onto BTY, IB-RS-2, and LFBK-α_V_β_6_ cells. Each cell tube (*n* = 4 or 5 tubes per cell line) was washed with 2 mL sterile PBS and then inoculated with 0.2 mL of sample. The tubes were incubated stationary at 37°C for 30 min and after incubation, each tube received 2 mL MEM. Cell culture tubes were incubated at 37°C with rotation and examined microscopically for CPE every 24 h up to a maximum of 96 h. All isolated samples were then characterized by antigen ELISA (5) and VP1 sequencing.

### Matrix Cytotoxicity

To determine whether sample matrices have an effect on cell monolayers, undiluted bovine serum, milk, probang, and whole blood were inoculated onto BTY, IB-RS-2, ZZ-R 127, and LFBK-α_V_β_6_ cells, and a 10% fecal suspension (SVDV sample type) was inoculated on IB-RS-2 and LFBK-α_V_β_6_ cells. Each cell tube (*n* = 4 per cell line) was washed with 2 mL sterile PBS and then inoculated with 0.2 mL of the matrix. The tubes were incubated stationary at 37°C for 30 min. After incubation, the monolayers were washed at least 3 times with 2 mL sterile PBS before adding 2 mL MEM to each tube. Cell culture tubes were incubated at 37°C with rotation for 72 h, and then examined microscopically for cytotoxicity.

### SVV, VESV, and VSV

BTY, IB-RS-2, ZZ-R 127, and LFBK-α_V_β_6_ cells (*n* = 3 tubes per cell line) were assessed for their ability to propagate SVV, VESV, and VSV. Cell culture tubes were washed with 2 mL sterile PBS and each tube received 2 mL MEM. Tubes were inoculated with 0.2 mL of SVV, VESV, or VSV, and then incubated at 37°C with rotation for 72 h. After 72 h, the cell monolayers were examined microscopically for CPE.

### Statistical Analysis

Average viral titers for FMDV/A/IRN/24/2012 and FMDV/O/KUW/4/2016 amongst BTY, IB-RS-2, ZZ-R 127, and LFBK-α_V_β_6_ cells were compared using Kruskal-Wallis and *post-hoc* Dunn's multiple comparisons tests. Average viral titers for SVDV/UKG/77/80 between IB-RS-2 and LFBK-α_V_β_6_ were compared using the Mann-Whitney test. Where epithelial suspensions were tested amongst cell lines and provided a single data point, the differences in sensitivity to FMDV for ZZ-R 127 and LFBK-α_V_β_6_ were compared to BTY cells independently, using paired *t*-tests. Statistical analysis was not performed on the O/CATHAY sensitivity data due to the low number of isolates detected. Statistical analyses were performed on log transformed titer values using Graphpad Prism 8.1.2. *P* < 0.05 were considered significant.

## Results

### Longevity of Sensitivity to FMDV and SVDV

**Figure 1:**
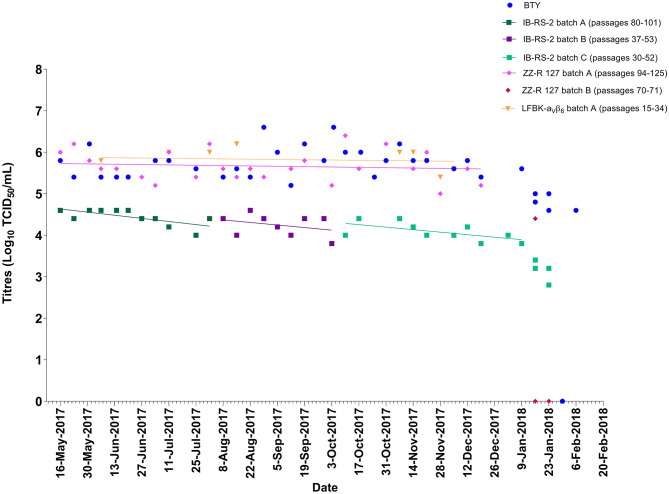
Titers of FMDV A/IRN/24/2012 epithelium suspension tested on BTY, IB-RS-2, ZZ-R 127, and LFBK-α_V_β_6_ cells. The lines represent the trend of titers over time. No trendline is present for BTY cells as these are independent, weekly batches.

**Figure 2:**
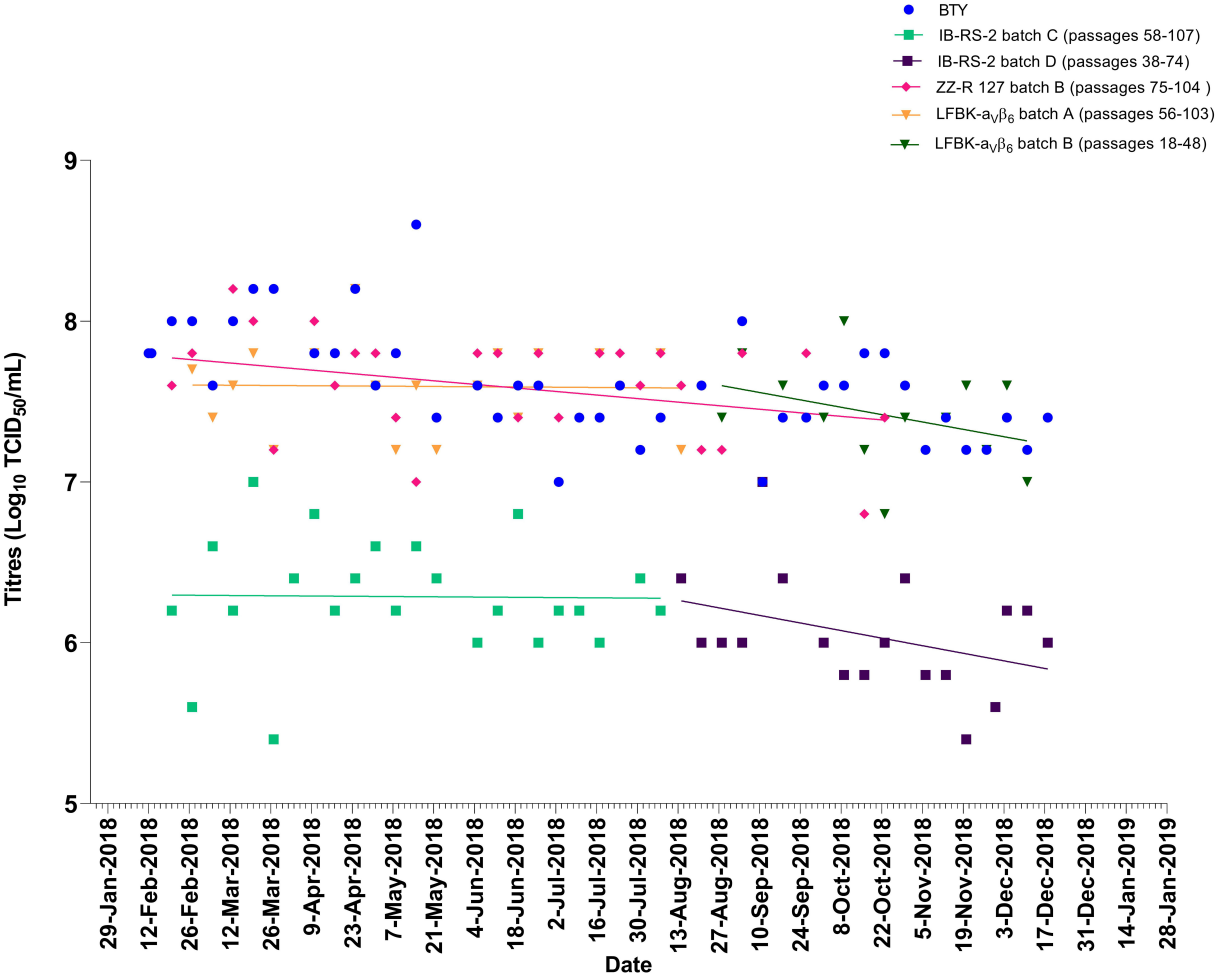
Titers of FMDV O/KUW/4/2016 epithelium suspension tested on BTY, IB-RS-2, ZZ-R 127, and LFBK-α_V_β_6_. The lines represent the trend of titers over time. No trendline is present for BTY cells as these are independent, weekly batches.

Over a 19-month period, weekly titrations were performed on BTY, IB-RS-2, ZZ-R 127, and/or LFBK-α_V_β_6_ cells using FMDV A/IRN/24/2012 (Figure 1) or O/KUW/4/2016 (Figure 2) epithelium suspensions; not all cell types were available each week, resulting in minor gaps in testing. The viral titers obtained from the weekly batches of BTY cells were within ±1 log_10_. The longevity of sensitivity for IB-RS-2 cells was inconsistent between batches (range 9–35 weeks), and in each case, the cells gradually lost their sensitivity over time, as evident by the decreasing titers (Figure 1). Once a batch of IB-RS-2 cells lost sensitivity, a new batch was revived for testing. The LFBK-α_V_β_6_ and ZZ-R 127 cell lines remained sensitive to FMDV for >100 passages, although the LFBK-α_V_β_6_ cells underwent senescence at passage 105 and the batch of cells were replaced. The two batches of ZZ-R 127 cells were replaced (after 33 and 43 weeks) before a noticeable decline in sensitivity to FMDV could be observed.

The average viral titers of epithelium suspensions FMDV/A/IRN/24/2012 (mean ± standard deviation; BTY; 5.9 ± 0.3, ZZ-R 127; 5.8 ± 0.4, LFBK-α_V_β_6_; 5.9 ± 0.3 and IB-RS-2; 4.3 ± 0.3 Log_10_ TCID_50_/mL) and FMDV/O/KUW/4/2016 (BTY; 7.9 ± 0.3, ZZ-R 127; 7.7 ± 0.3, LFBK-α_V_β_6_; 7.7 ± 0.3 and IB-RS-2; 6.4 ± 0.4 Log_10_ TCID_50_/mL) were significantly different by cell type (*p* < 0.001). For both FMDV A/IRN/24/2012 and O/KUW/4/2016, the sensitivity of ZZ-R 127 and LFBK-α_V_β_6_ cells were comparable to BTY cells; however, the sensitivity of the IB-RS-2 cells was significantly lower than BTY, ZZ-R 127 and LFBK-α_V_β_6_ cells (*p* < 0.0001).

**Figure 3:**
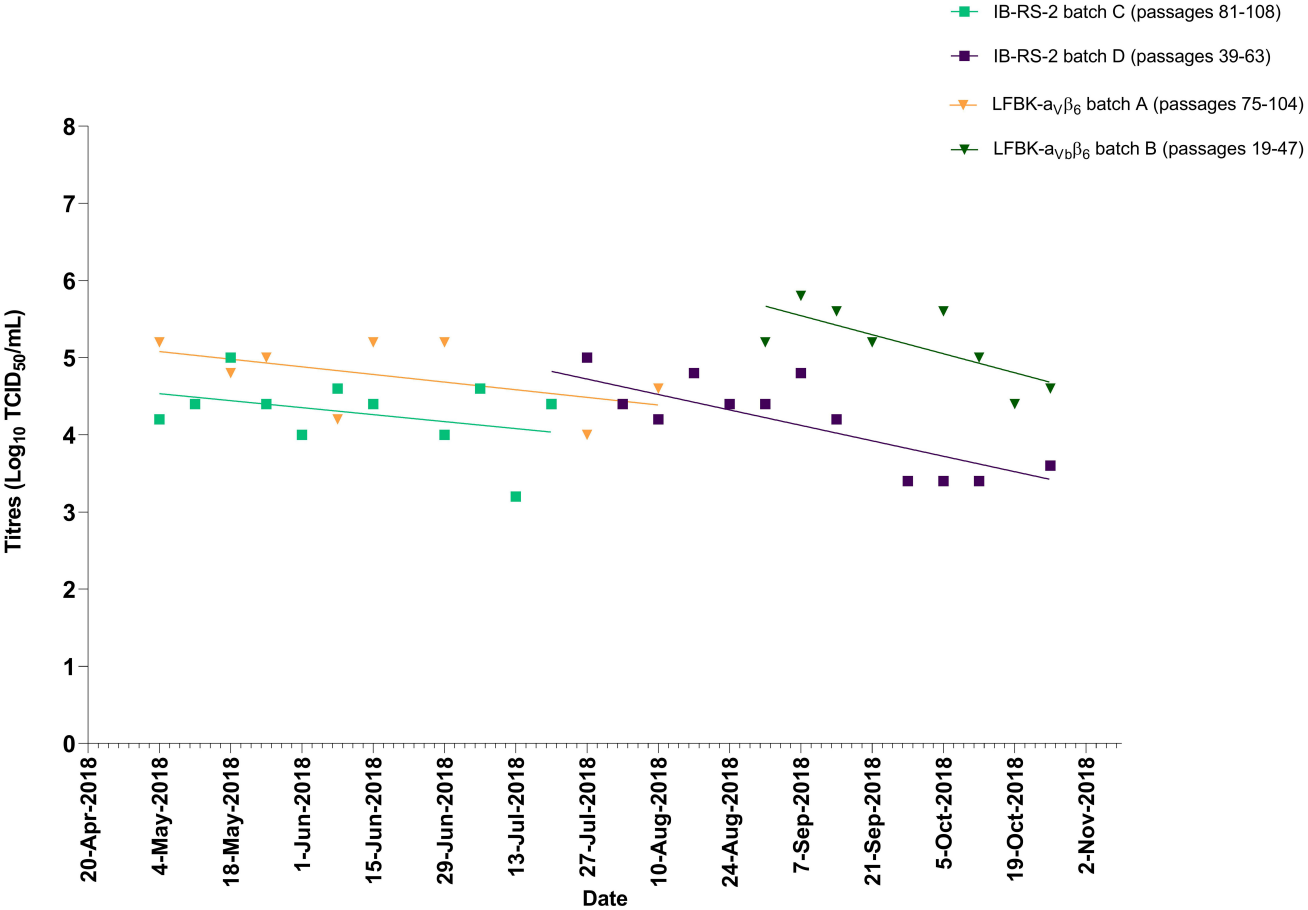
Titers of SVDV/UKG/77/80 epithelium suspension tested on IB-RS-2 and LFBK-α_V_β_6_. The lines represent the trend of titers over time.

Over an 8-month period, weekly titrations were performed on IB-RS-2 and/or LFBK-α_V_β_6_ cells using SVD/UKG/77/80 (Figure 3). IB-RS-2 and LFBK-α_V_β_6_ cells were not available each week, hence the minor gaps (maximum of 4 weeks) in testing. IB-RS-2 and LFBK-α_V_β_6_ cells remained sensitive to SVDV for >100 passages. The LFBK-α_V_β_6_ cells lost sensitivity to SVDV at passage 104, as indicated by the lack of viral titer (Figure 3). The LFBK-α_V_β_6_ trend lines indicate that titers decreased overtime similar to the IB-RS-2 cell line. The average titer for the SVDV/UKG/77/80 epithelium suspension was significantly higher in the LFBK-α_V_β_6_ than IB-RS-2 cells (*p* < 0.001; 5.2 ± 1.2 and 4.5 ± 0.5 Log_10_ TCID_50_/mL, respectively),

### Detection of FMDV in Diagnostic Epithelium Suspensions

**Figure 4:**
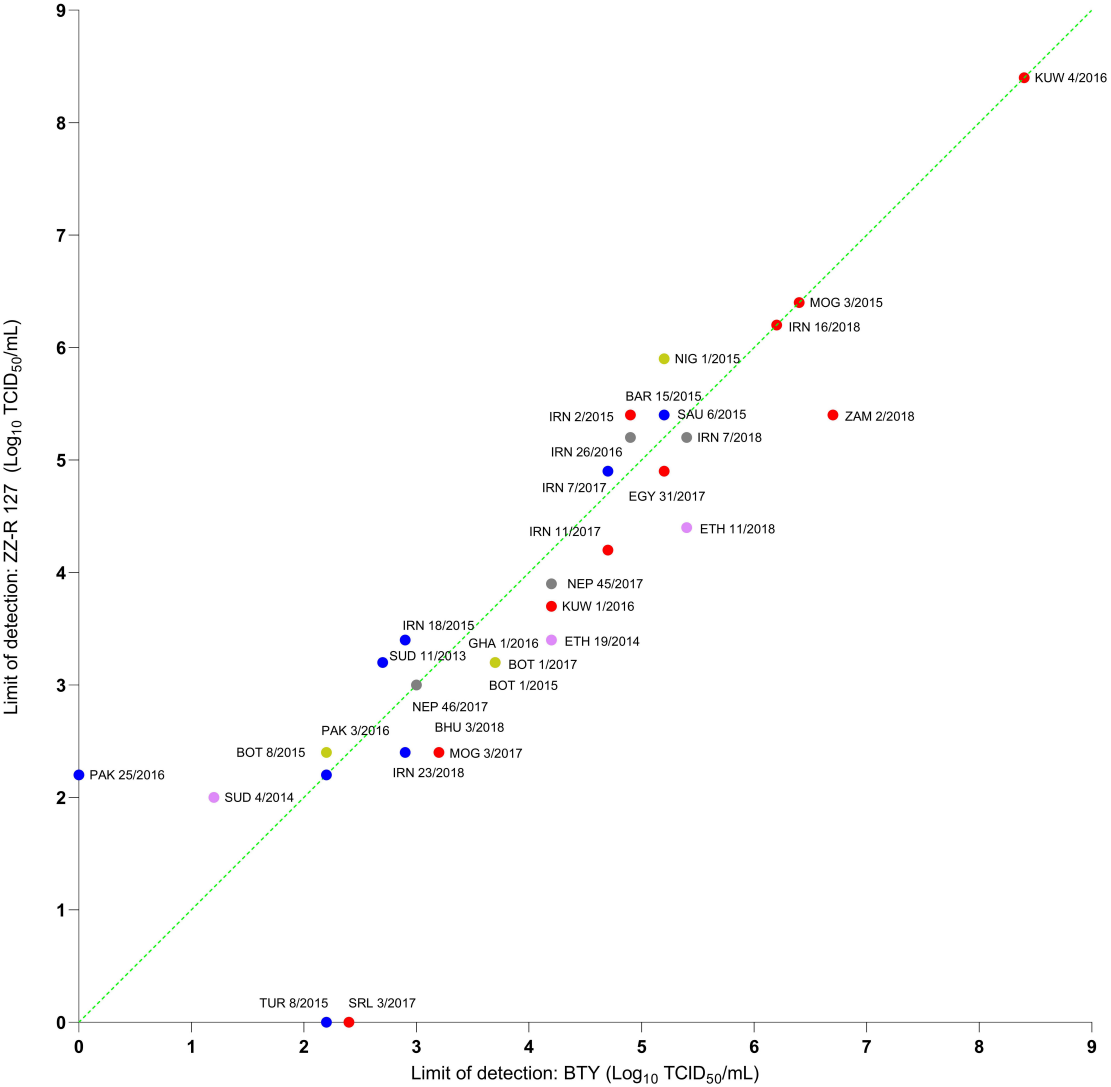
Titers of epithelium suspensions tested on primary BTY cells and ZZ-R 127 cells. Samples are color coded based on FMDV serotype as follows: O, A, SAT 1, SAT 2, and Asia 1. The dotted green line indicates where the limit of detection was identical between the cells; samples with data points above the line indicate a lower limit of detection in ZZ-R 127 and samples with data points below the line indicate a lower limit of detection in BTY cells.

**Figure 5:**
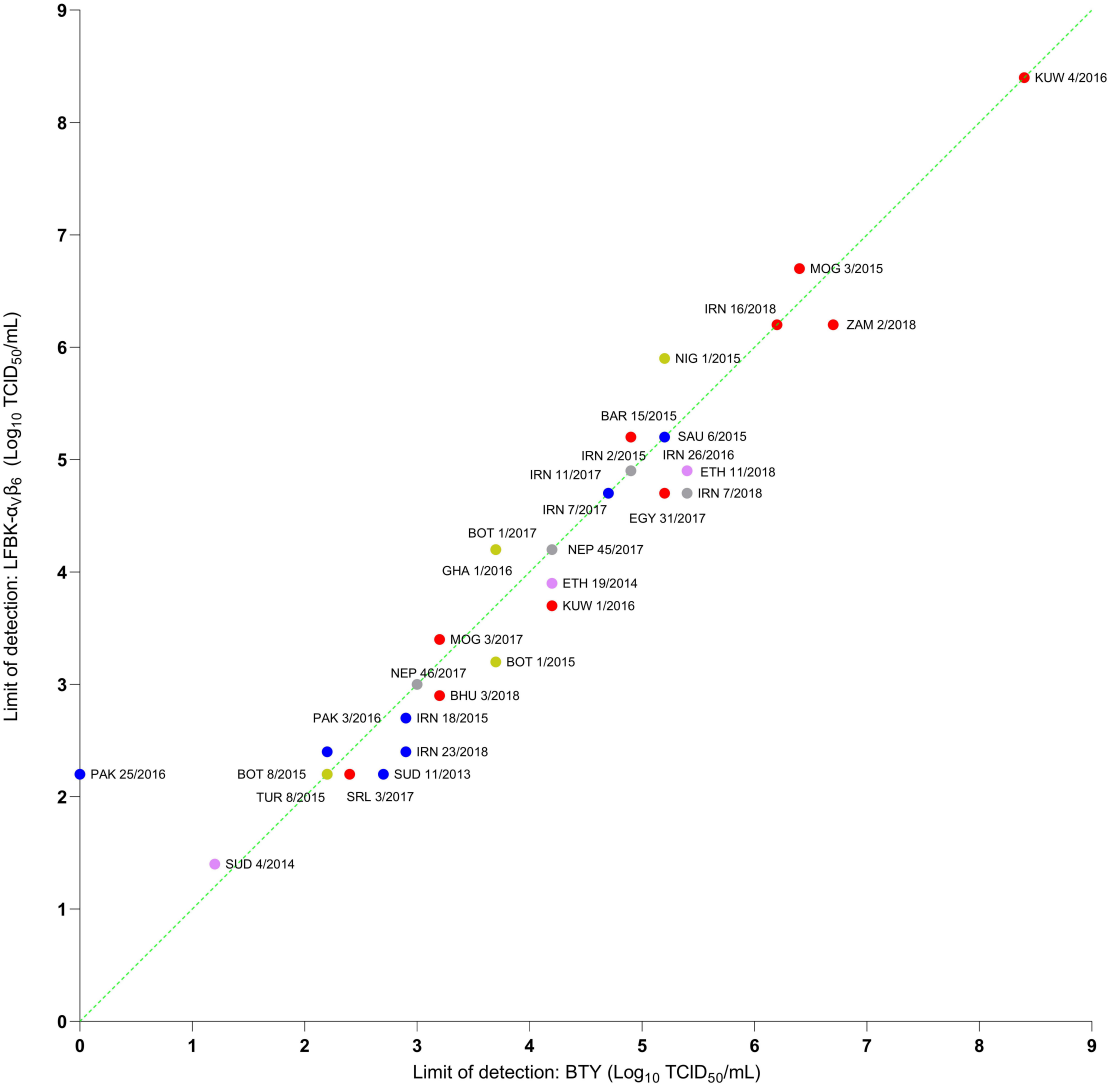
Titers of epithelium suspensions tested on primary BTY cells and LFBK-α_v_β_6_ cells. Samples are color coded based on FMDV serotype as follows: O, A, SAT 1, SAT 2, and Asia 1. The dotted green line indicates where the limit of detection was identical between the cells; samples with data points above the line indicate a lower limit of detection in LFBK-α_v_β_6_ and samples with data points below the line indicate a lower limit of detection in BTY cells.

Thirty-two epithelium suspensions were titrated using BTY, ZZ-R 127, and LFBK-α_V_β_6_ cells and the limit of detection compared by calculating the relative viral titer generated in these different cell systems. In the majority of samples tested (29 of 32), the analytical limit of detection for the ZZ-R 127 and LFBK-α_V_β_6_ was comparable to that of the primary BTY cells (Figures 4, 5). Overall, there was no significant difference in analytical sensitivity between BTY and either ZZ-R 127 or LFBK-α_V_β_6_ (*p* > 0.05). Nonetheless, the LFBK-α_V_β_6_ cells showed a high degree of diagnostic capability by successfully propagating virus from all epithelium suspensions tested, whereas three viral suspensions were unable to replicate in either the BTY or ZZ-R 127 cells. A/PAK/25/2016 was undetected in BTY cells, despite originally being isolated in this cell type, and A/TUR/8/2015 and O/SRL/3/2017 were undetected in ZZ-R 127 cells.

Eight O/CATHAY epithelium suspensions were titrated using IB-RS-2, ZZ-R 127, and LFBK-α_V_β_6_ cells. Despite the eight suspensions being originally isolated in IB-RS-2 cells during diagnostic testing at the time of submission, only one epithelium suspension (HKN/5/2016) was able to generate a titer in IB-RS-2 cells during repeat testing in this study. Although the epithelium suspensions had been stored at −80°C since use, it is likely that the viral titer of the samples had decreased during storage and through freeze-thawing. The majority of these samples (6 of 8) did not cause CPE in the ZZ-R 127 cells; only two epithelium suspensions, HKN/11/2017 and HKN/5/2016, generated viral titers. In contrast, all eight samples replicated in LFBK-α_V_β_6_ cells. The LFBK-α_V_β_6_ cells had increased sensitivity to the O/CATHAY topotype in comparison to IB-RS-2, with higher titers observed for all eight epithelium suspensions correlating to a lower limit of detection.

Initially, six porcine epithelium suspensions, negative for virus isolation using BTY and IB-RS-2 cells but positive for FMDV genome, were inoculated onto LFBK-α_V_β_6_ cells. From these six suspensions, two viruses were isolated in LFBK-α_V_β_6_ cells (Table 2). A further 20 porcine epithelium suspensions originating from Hong Kong SAR were inoculated onto BTY, IB-RS-2, and LFBK-α_V_β_6_ cells in parallel at the time of submission. Out of these 20 suspensions, 12 viruses were isolated in LFBK-α_V_β_6_ cells only. In total, viruses were isolated in 14/26 samples using LFBK-α_V_β_6_ cells, which otherwise would not have been undetected, and were subsequently characterized by antigen ELISA and VP1 sequencing as O/CATHAY topotype.

**Table 2:** Number of CPE positive replicates for BTY, IB-RS-2, and LFBK-α_V_β_6_ post-inoculation with porcine samples received from Hong Kong (*n* = 26).

**Sample reference**	**BTY**	**IB-RS-2**	**LFBK-α_V_β_6_**	**FMDV 3D C_T_ values**
HKN 5/2017^*^	0/5	0/5	1/4	28.68
HKN 2/2018^*^	0/5	0/5	0/5	25.57
HKN 3/2018^*^	0/5	0/5	0/5	20.22
HKN 4/2018^*^	0/5	0/5	2/5	25.72
HKN 7/2018^*^	0/5	0/5	0/5	32.91
HKN 9/2018^*^	0/5	0/5	0/5	28.30
HKN 10/2018	0/5	0/5	0/5	27.91
HKN 11/2018	0/5	0/5	5/5	21.53
HKN 12/2018	0/5	0/5	3/5	38.37
HKN 13/2018	0/5	5/5	5/5	24.94
HKN 14/2018	0/5	0/5	5/5	No C_T_
HKN 15/2018	0/5	0/5	5/5	33.94
HKN 16/2018	0/5	0/5	5/5	34.51
HKN 17/2018	0/5	0/5	4/5	39.08
HKN 18/2018	0/5	0/5	3/5	32.92
HKN 19/2018	0/5	0/5	0/5	35.89
HKN 20/2018	0/5	5/5	5/5	18.83
HKN 21/2018	0/5	1/5	5/5	34.43
HKN 22/2018	0/5	0/5	0/5	36.35^†^
HKN 23/2018	0/5	5/5	5/5	22.71
HKN 1/2019	0/5	2/5	4/4	33.05
HKN 2/2019	0/5	0/5	4/4	35.99
HKN 4/2019	0/5	0/5	4/4	33.02
HKN 5/2019	0/5	0/5	4/4	No C_T_
HKN 6/2019	0/5	0/5	3/4	34.76
HKN 7/2019	0/5	0/5	4/4	36.36^†^

*Samples were received to the WRLFMD between 2017 and 2019. All isolated viruses were confirmed as O/CATHAY topotype by VP1 sequencing. FMDV 3D qRT-PCR C_T_ values are included for comparison and are an average of two replicates*.

* *LFBK-α_V_β_6_ inoculated independently from BTY and IB-RS-2 cells*.

† *Sample provided a C_T_ value in only one replicate*.

### Effects of Sample Matrices

No cytotoxicity was observed in the BTY cultures for any of the matrices tested. Cytotoxicity was not observed in IB-RS-2, ZZ-R 127, and LFBK-α_V_β_6_ cells for serum, probang fluid and milk; however, whole blood caused cytotoxicity in all four replicates of each of the continuous cell lines where patches of adherent cells were stripped from the tube surfaces. No cytotoxicity was caused by the 10% pig fecal suspension, which was inoculated onto IB-RS-2 and LFBK-α_V_β_6_ cells.

### Susceptibility to Other Vesicular Viruses

No CPE was observed in BTY cells 72 h after inoculation with SVV, VESV, and VSV, indicating BTY cells cannot propagate these viruses (Table 3). The ZZ-R 127 cells were able to propagate VESV and VSV, producing CPE in each of the replicates, whereas SVV was unable to propagate as indicated by the lack of CPE. The IB-RS-2 and LFBK-α_V_β_6_ cells were able to support the replication of all three vesicular viruses tested.

**Table 3:** Number of replicates per cell line with CPE after inoculation with SVV, VESV, and VSV.

**Cell line**	**BTY**	**ZZ-R 127**	**LFBK-α_V_β_6_**	**IB-RS-2**
SVV-MN-88-36695	0/3	0/3	3/3	3/3
VESV-K54	0/3	3/3	3/3	3/3
VSV-New Jersey	0/3	3/3	3/3	3/3

## Discussion

In this study, we have shown that the ZZ-R 127 and LFBK-α_V_β_6_ cell lines were susceptible to FMDV for >100 passages (Table 4), and the analytical limit of detection of these cell lines was comparable to primary BTY cell cultures. In comparison, the sensitivity of the IB-RS-2 cell line was significantly lower than ZZ-R 127, LFBK-α_V_β_6_, and BTY cells. Our results highlight the known decreased sensitivity of these cells to FMDV (8). The IB-RS-2 cells lost sensitivity over time, but the ZZ-R 127 and LFBK-α_V_β_6_ cells remained consistently sensitive during progressive sub-culturing (Figures 1, 2). These data confirmed previous studies that reported the ZZ-R 127 and LFBK-α_V_β_6_ cell lines were highly sensitive to FMDV (10, 11, 21).

**Table 4:** Summary comparing the results among BTY, ZZ-R 127, LFBK-α_V_β_6_, and IB-RS-2.

		**Cell type**			
		**BTY**	**ZZ-R 127**	**LFBK-α_V_β_6_**	**IB-RS-2**
Duration of FMDV sensitivity		3–4 weeks per batch	>100 passages	>100 passages	>100 passages
Duration of SVDV sensitivity		ND	ND	>100 passages	>100 passages
Sensitivity of cell lines	FMD/A/IRN/24/2012	5.8	5.7	5.8	4.3
	FMD/O/KUW/4/2016	7.9	7.7	7.7	6.4
(Avg. Log_10_ TCID_50_/mL)	SVD/UKG/77/80	ND	ND	5.2	4.5
Detected FMDV epithelium suspensions		31/32	30/32	32/32	ND
Detected O/CATHAY epithelium suspensions		ND	2/8	8/8	1/8
Detected O/CATHAY diagnostic submissions		0/26	ND	19/26	5/26
Susceptibility to other vesicular viruses		None	VESV, VSV	VESV, VSV, SVV	VESV, VSV, SVV
Matrices causing cytotoxicity		None	Whole blood	Whole blood	Whole blood

*ND, not done*.

In comparison to IB-RS-2, our data highlight the increased longetivity of the ZZ-R 127 and LFBK-avb6 cell lines to support FMDV replication. We anticipate that these findings will be broadly transferable to other laboratories, but specific cell batches and culture conditions may influence these results. Therefore, prior to use for routine diagnostics, we recommend that cell sensitivity should be monitored using dilutions of a well-characterized reference FMD virus.

When the diagnostic sensitivities of ZZ-R 127 and LFBK-α_V_β_6_ cells were assessed using a range of FMDV field strains, the LFBK-α_V_β_6_ cells detected all 32 samples (Table 4) whereas, the ZZ-R 127 and BTY cells detected 30 and 31 samples, respectively. The 32 samples were selected to encompass multiple FMDV serotypes and topotypes (serotype O *n* = 20, A *n* = 8, SAT 1 *n* = 4, SAT 2 *n* = 3, and Asia 1 *n* = 5). Serotype C was not included in this study because it is not known to be circulating; it was last detected in Kenya and Brazil in 2004 (26). No SAT 3 epithelium suspensions were tested due to limited availability of material.

Cell lines of porcine origin are utilized for the detection of pig-adapted FMDV topotypes (e.g., O/CATHAY) and other porcinophilic vesicular viruses. Here, we demonstrated that LFBK-α_V_β_6_ cells were sensitive to SVDV for >100 passages, and provided a significantly higher limit of detection than the IB-RS-2 cell line (Figure 3). The LFBK-α_V_β_6_ cells were also highly susceptible to infection with isolates from the pig-adapted O/CATHAY FMDV topotype. Overall, our data demonstrated that the LFBK-α_V_β_6_ cell line is more sensitive to FMDV and SVDV than IB-RS-2, possibly because of the constitutive expression of the bovine α_V_β_6_ integrin receptor. The only potential disadvantage of the LFBK-α_V_β_6_ cell line is that they are contaminated with a non-cytopathic BVDV (Rodriguez LL, personal communication, 2019).

The most common sample type submitted to the WRLFMD for the diagnosis of vesicular diseases is epithelium from vesicular lesions. FMD virus can be isolated from other samples types, including whole blood, serum, milk, probang fluid, and feces; however, these matrices can cause detrimental effects to cells, and thus compromise virus isolation. Of the matrices tested, primary BTY cells were the most robust, in that no cytotoxicity was observed. No cytotoxicity was observed in ZZ-R 127 and LFBK-α_V_β_6_ cells after inoculation with serum and probang fluid, supporting the findings that these cell types can be used to isolate FMDV from serum and probang of experimentally infected animals (21). The only matrix that caused cytotoxicity was the undiluted bovine whole blood, which stripped patches of cells from the monolayers of IB-RS-2, ZZ-R 127, and LFBK-α_V_β_6_.

While virus isolation is a sensitive diagnostic test, the observation of CPE is not virus specific. There are several notifiable diseases that are clinically indistinguishable from FMDV, such as VSV, VESV, and SVV, which cause similar CPE in cell culture. Of the four cell types tested, IB-RS-2 and LFBK-α_V_β_6_ cells were the most versatile, in that VSV, VESV, and SVV were all able to replicate, confirming previous results that the LFBK-α_V_β_6_ cells are capable of propagating VESV and VSV (11). Cell lines currently available for the isolation of SVV include swine testis cells, porcine kidney, IB-RS-2 and BHK (27). To our knowledge, this is the first time LFBK-α_V_β_6_ cells have been identified as a resource for the isolation of SVV.

As mentioned, primary BTY cells are accepted as the most sensitive cell culture for the isolation of FMDV, but their preparation is expensive and labor intensive (23). Hence, diagnostic laboratories would benefit from a continuous cell line with the same sensitivity as primary BTY cells. Although sensitivity comparisons have been performed between BTY and ZZ-R 127 (10) and between ZZ-R 127 and LFBK-α_V_β_6_ cells (21), this is the first study to compare LFBK-α_V_β_6_ and primary BTY cells. The results indicate that both ZZ-R 127 and LFBK-α_V_β_6_ cell lines are suitable alternatives to BTY cells for the isolation of FMDV. Furthermore, the LFBK-α_V_β_6_ cells have multiple advantages in that the cells grow quickly in cell culture, remained stable for >100 passages, and were able to support growth of all the other vesicular viruses tested. This contrasts with the ZZ-R 127 cells which grow slowly and were not able to support growth of SVV.

Rapid and accurate diagnosis underpins the control of FMDV. Although virus isolation is not a rapid diagnostic test (i.e., it can take 1–6 days to isolate a virus), it is necessary for downstream testing, such as vaccine matching. At WRLFMD, FMD serotype is most commonly determined using a polyclonal antigen ELISA (5), or a monoclonal antigen ELISA (28). Epithelium suspensions prepared from clinical samples can be tested directly on an ELISA, but only approximately a third of samples submitted to the WRLFMD contain the concentration of viral antigen needed for detection [e.g., minimum concentration of 1–2 ng/mL of virus antigen for detection with the polyclonal antigen ELISA (29)]. In addition, samples such as blood, serum, probang fluid, milk, and feces cannot be tested directly on ELISA. Consequently, clinical samples such as these must be isolated in cell culture before testing with a serotyping antigen ELISA.

Recently, lineage-specific real-time RT-PCR assays have been developed to circumvent the need for virus isolation and the handling of “live” virus (30–33). However, due to the diversity of FMDV topotypes and the rapid mutation rate of the RNA genome (34), these assays need to be tailored to geographic regions and require ongoing monitoring of sensitivity. Additionally, these assays are “dead end tests,” as the material produced cannot be used for downstream testing, such as vaccine matching. Although serotyping real-time RT-PCRs have advantages, these assays cannot yet replace virus isolation.

Currently, the use of sensitive cell cultures are required for testing vaccine efficacy to a particular field strain (25). The virus neutralization test requires the serial passage of an FMDV isolate to generate a high viral titer and is dependent on the use of continuous cell lines, such as BHK and IB-RS-2, to determine the ability of antibodies to neutralize “live” virus. While both ZZ-R 127 and LFBK-α_V_β_6_ cell lines represent suitable alternatives, it is expected that the LFBK-α_V_β_6_ cell line will undergo validation for virus neutralization tests in the WRLFMD due to their susceptibility to a wider range of FMDV strains, including the O/CATHAY topotype.

## Conclusions

In this study, the FMDV sensitivity of the ZZ-R 127 and LFBK-α_V_β_6_ cell lines were comparable to primary BTY cells, and significantly higher than the IB-RS-2 cell line (Table 4). In addition, the LFBK-α_V_β_6_ cells were significantly more sensitive to SVDV than the IB-RS-2 cells and exhibited a high diagnostic capability for detecting the O/CATHAY pig-adapted FMDV strain. Overall, ZZ-R 127 and LFBK-α_V_β_6_ cell lines have been confirmed as sensitive tools for FMDV diagnostic testing. The LFBK-α_V_β_6_ cells outperformed the IB-RS-2 throughout testing and therefore, have been identified as a highly sensitive porcine cell line for the routine detection of FMDV strains and porcinophilic vesicular viruses.

## Data Availability Statement

The raw data supporting the conclusions of this article will be made available by the authors, without undue reservation.

## Author Contributions

AG, VM, BW, and DK conceived the study. DK obtained funding. AG, VM, BW, MA, and EH performed virus titrations and inoculations. AG performed data interpretation and wrote the manuscript. All authors read and approved the manuscript content.

## Conflict of Interest

The authors declare that the research was conducted in the absence of any commercial or financial relationships that could be construed as a potential conflict of interest. The handling editor declared a past co-authorship with one of the author DK.
